# Hybrid Interfacial Transition Zone between Wet—On—Wet Casted Concrete—Microstructure and Mechanical Properties

**DOI:** 10.3390/ma15196511

**Published:** 2022-09-20

**Authors:** Klaudja Telhaj, Hans Hedlund, Andrzej Cwirzen

**Affiliations:** 1 Department of Civil, Environmental, and Natural Resources Engineering, Luleå University of Technology, 971 87 Luleå, Sweden; 2 Skanska AB, 405 18 Göteborg, Sweden

**Keywords:** hybrid concrete, interfacial transition zone, porosity, bond strength

## Abstract

The manufacture of elements containing two types of concrete allows for the minimization of the amount of Portland cement by matching the properties of concrete with local structural and durability requirements. The most common production method of the hybrid element is wet–on–hard and wet–on–wet. Casting wet–on–dry is the most common approach while casting wet–on–wet has been used mostly for concrete overlays and screeds. The study focuses on the wet–on–wet method but is applied in the production of vertical and horizontal elements. Bond-behavior and micro properties of the wet–on–wet casting interface of ultra–high–performance concrete (UHPC)–normal strength concretes are investigated. The obtained results indicate the formation of a hybrid interfacial transition zone between the two types of casted concrete. The binder matrix located in this zone appeared to combine properties of both used concrete. Porosity, phase composition, and presumably also strength, changed gradually. Furthermore, despite significant differences in shrinkage, no microcracking or delamination was observed in that zone. The ultimate flexural and compressive strength of the produced elements were either equal to the stronger concrete or were higher than the weaker of the used concrete.

## 1. Introduction

The annual production of concrete exceeds 30 billion tons worldwide [1]. The majority of currently produced concrete is based on Portland cement, which manufacture is responsible for 5–8% of the total global CO_2_ emission [2,3]. Research in the field of civil engineering related to the reduction of CO_2_ emission and to the sustainability of structures has advanced significantly in recent years. A partial replacement of Portland cement with secondary cementitious material (SCM) can reduce the CO_2_ footprint. Typical SCMs include fly ash, blast furnace slag (BFS), and calcined clay [4,5,6]. Most concretes containing SCMs have certain limitations, for example, slower strength development, delayed setting time, or less durability. On the other hand, the Ultra–High–Performance Concrete (UHPC) has shown an excellent performance predominantly due to its very dense hardened matrix. Good durability and low maintenance requirement of structures made using UHPC be considered more cost-effective than comparable structures made from normal–strength concretes, especially when a long life span of the structure is crucial [7]. However, the high amount of cement leads to a high environmental impact, and the initial costs are regarded as a disadvantage, restricting its wider application [8].

Structural elements such as walls, slabs, and columns exposed to severe environmental conditions and high structural loads can be strengthened by combining UHPC with normal strength/ecological concrete. The combination could include, for example, concrete with a very low CO_2_ footprint, but having insufficient frost durability, with an external layer made of UHPC or HPC [9,10]. Bruhwiler, E., 2008 has also proposed different applications of UHPC to strengthen deteriorated bridges, cash barrier walls, and industrial floors [11]. Retrofitting existing structures with UHPC could limit the required maintenance thus lowering cost [12]. Two casting methods can be used to manufacture a structure made of hybrid concrete, i.e., wet–on–dry and wet–on–wet. The difference between these two methods is the state of the substrate, which can be hardened and even-aged, or fresh [13]. In both manufacturing methods, the interfacial transition zone (ITZ) forming between the two casted concrete is the key factor that determines the bond strength, mechanical properties, and durability of the structure. Wet–on–dry method is commonly used for repairs or strengthening of existing concrete structures. Bond strength depends on the hydration of the fresh concrete and the surface preparation of the existing concrete and its moisture content [14,15,16,17]. Varga et al., 2018 [18] concluded that the increased moisture content of the surface of existing concretes appeared to improve the consolidation enhanced the hydration, and lowered the porosity of the ITZ. Kothari et al., 2020 [19] studied the ITZ formed between the water jetted surface of a normal strength concrete (NSC) and ultra-high performance concrete (UHPC) which had a thickness of <20 µm. The measured bond strength was higher than the tensile strength of the NSC concrete. The failure in the pull-out test always occurred in the NSC [19]. Others also observed a very homogenous microstructure of the ITZ between NSC and UHPC. No cracks were observed, even after the shear strength testing was reported by Tayeh et al. [20], when the substrate surface was prepared by sandblasting. However, in the wet–on–dry method the required surface preparation tends to elongate the manufacturing time and an additional variability of the ITZ bond [21]. In the case of the wet–on–wet method, there is no need for additional surface preparation. This reduces the manufacturing time but also enhances the bond strength between the casted concretes.

Previous studies on the wet–on–wet method focus on the mechanical properties [10,22,23]. The arrangement of the layers and the thickness of the bottom layer affected the flexural strength of the hybrid concrete. Yalçınkaya [24] investigated the combination of the UHPC with a steel reinforcement with normal mortar (NM) and self–compacting mortar (SCM)which resulted in higher load carrying capacity. The interface of these hybrid concretes did not sustain any damage. L. Hussein [25] investigated the flexural strength of the hybrid concrete cast as wet–on–wet, consisting of UHPFRC and NC/HSC and being layered horizontally. The addition of various amounts of fibers to the bottom casted UHPFRC layer increased the flexural capacity. A splitting test was conducted to evaluate the bond strength between UHPFRC and NC/HSC. The bond failure occurred along the interface. Only two specimens containing 1.5 vol.% of fibers sustained the failure in the weaker NC and HSC concretes. In both tests, the bond strength between layers casted as wet–on–wet was significantly higher compared to the reference beams made of NC and HSC [25]. The microstructure of the hybrid concrete (UHPFRC–NC) exposed to a chloride and sodium sulfate environment was also investigated in that research by L. Hussein [26,27]. The wet and dry cycles were repeated for 600 days for the chloride exposure and 180 days for the sodium sulfate. The interface integrity of all specimens was sustained despite these extreme exposures. The wet–on–wet casting interface of the hybrid concrete has shown good bond strength and good durability.

Furthermore, more research and investigation should be added to understand how the hydration process affects the interface microstructure and how it is reflected in the macro bonding performance of the hybrid concrete casted on wet–on–wet.

As shown the number of studies related to the wet–on–wet method is rather limited. Understanding the hydration process that takes place in the interface is important due to its effects on the interface. The focus of this study was the determination of the phase composition and microstructure of the binder matrix forming in the interface zone between two-cast wet–on–wet concretes. Additionally, their effect on selected mechanical properties of elements made of hybrid concrete was evaluated.

## 2. Methods

Six combinations of four types of concretes have been used. Including normal strength concrete (NSC), blast furnace slag concrete (BFSC), and ultra–high–performance concrete with (UHPFRC) and without fibers (UHPC). Test samples were cast in vertical or horizontal layers.

### 2.1. Materials

Portland cement type CEM I 42.5N (Anläggningscement) used was produced by “Cementa” (Skövde, Sweden). Ground granulated blast furnace slag (BFS), type merit 5000 (Merox, Oxelösund, Sweden) used was provided by SweCem (Helsingborg, Sweden). The maximum granite aggregate size used was 8 mm. The OPC to BFS ratio was 50:50. The UHPC concrete contained Portland cement CEMI 42.5N, condensed micro-silica 920D from Elkem (Oslo, Norway), limestone powder “Nordkalk Limus 40” from Nordkalk AB, Norquartz 45 from Sibelco Nordic (Lillesand, Norway) as well as micro sand B15 (150 µm) and B35 (350 µm) provided by Baskarpsand AB (Habo, Sweden). A polycarboxylate-based superplasticizer type “MasterGlenium ACE 30” from BASF (Rosersberg, Germany) was added to all mixes. Steel fibers having lengths of 7 mm and 13 mm were provided by Krampe Harex-Germany (Hamm, Germany). The chemical composition of dry materials used is determined by ICP–SFMS (Inductively coupled plasma mass spectroscopy-sector field) as shown in Table 1.

### 2.2. Sample Preparation

The mixed proportion of concretes used is summarized in Table 2. The w/c ratio used for normal strength concrete was 0.4, 0.45, and 0.65 for the C2, N1, and N2, respectively. The cement content varied from 360–400 kg/m^3^. In the case of the BFSC, a replacement of 50% by weight of Portland cement is made with slag. The ultra-high performance contains 680 kg/m^3^ Portland cement and had a w/c = 0.3. In the UHPFRC, 20% of steel fibers by cement weight are added to the mix composition. Based on the proportion of fine grains in each of the mix compositions without changing the w/c ratio, different amounts of superplasticizer were adjusted to reach the desired workability [9,28]. All concrete mixes were produced using an 8-L “Hobart” type mixer. The mixing procedure varied depending on the produced concrete type. NSC and BFSC included the mixing of all dry components for 3 min, followed by the addition of water and superplasticizer and mixing for another 2 min. The mixing procedure for the UHPC and UHPFC concretes was slightly different. It consisted of mixing all dry components for 5 min, addition of water and superplasticizer, and an additional 5 min of mixing. Steel fiber with a length of 7 or 13 mm was added to the UHPFC mix and mixing continued for another 5 min. The total mixing time varied between 10 to 15 min.

**Figure 1:**
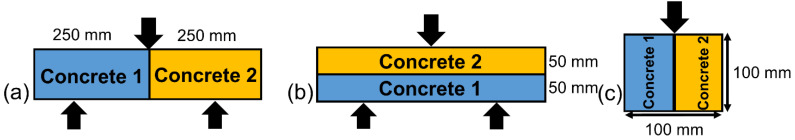
Used test specimen: (**a**) beam with the vertical arrangement, (**b**) beam with horizontal arrangement of layers, (**c**) cube with vertical arrangements.

Two concretes to be cast into the hybrid element were prepared almost at the same time and left stationary until placing in formwork occurred. Molds were marked to define the heights of the bottom and upper layers in a horizontal arrangement and the length in the vertical as shown in Figure 1. Samples had a dimension of 100 × 100 × 500 mm^3^ and 100 × 100 × 100 mm^3^.

The vertical arrangement consisted of two concretes poured simultaneously against a removable plastic lifting plate. The plate was removed after the completion of pouring. No vibration was used after the plate removal. The horizontal arrangement was cast in two layers without using any separating plate. Since the setting of UHPC was longer than NC [29], the hybrid concrete was casted upside down. First, the NC or BFSC layer was poured and compacted, and then the UHPC layer was poured immediately on top. Additionally, in the horizontal arrangement, no vibration was employed during the entire preparation process.

Six types of combinations of different types of concrete were made. To mark the hybrid concrete, the following symbols shown in Table 3 were used. All samples were cured in sealed conditions in the mold for 24 h at room temperature (20 ± 2 °C) followed by water curing until the age of 28 days.

### 2.3. Testing Methodology

The rheological properties of fresh concrete were evaluated by using a slump flow test by following the SS-EN 12350-8:2019 standard [30].

The microstructure of the ITZ was analyzed using a digital microscope and Scanning Electron Microscope (SEM)–type Jeol JSM-IT100 (JOEL Ltd., Tokyo, Japan) coupled with energy-dispersive spectrometry (EDS) from Bruker (Bruker Corporation, Billerica, MA, USA). For the analysis, a part of the interface was cut from 28-day test cubes, where the examined surface was perpendicular to the top surface of the cube [31]. The core of the specimen was immersed in isopropanol alcohol for five days to stop the hydration process. Later, samples were stored in a desiccator for seven days followed by impregnation under vacuum with a low viscosity epoxy resin. Polishing was completed using grinding plates and diamond spray having particle sizes of 9, 3 and 1 µm following a procedure described elsewhere [32]. Images were taken using a magnification of 55× for the digital microscope and 150×, 1000×, and 4000× for the SEM. The SEM images were captured alongside the ITZ in a backscattered electron mode (BSE). At 150× magnification, four images located in a row were captured on each side of the interface. The total length of the analyzed interface was 3300 µm and the width was 1800 µm. Images were “stitched” together and processed using the Image J analytical software ver.1.53c produced by Wayne Rasband (Bethesda, MD, USA). A similar procedure was applied to 12 images obtained at 1000× magnification. The total length of the analyzed interface zone was approximately 1300 µm and 475 µm wide. Additionally, SEM-EDS analysis was performed at 4000× magnification in locations which were selected based on the gray level identified as the C-S-H phase. The used procedure followed the method described elsewhere [32,33]. Variation of the Ca/Si atomic ratio was determined versus the distance from the visually estimated transition line. The analyzed area was divided into five zones, each having a width of 20 µm. In each zone, five areas were selected, and 20 spot analyses were performed for each area. Average Ca/Si atomic ratios were calculated. The SEM acceleration voltage was 15 kV, the electron beam current was 50 mA, and the chamber vacuum was 30 Pa. 50,000 counts per each analysis were used for the SEM-EDS.

**Figure 2:**
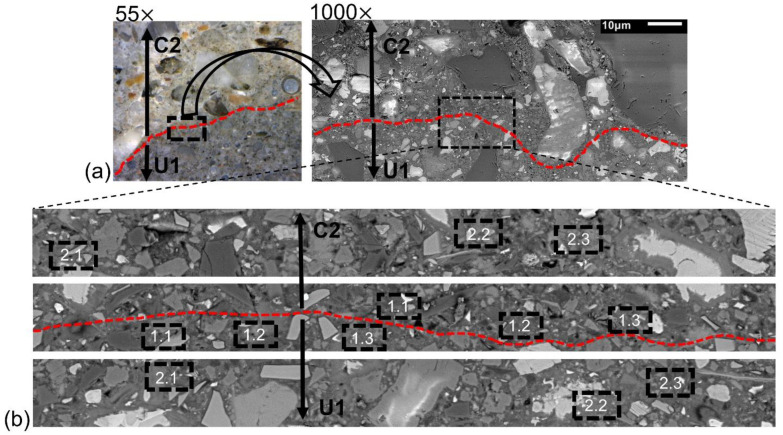
(**a**) Digital microscope image and “stitched” BES-SEM image with the rectangular area selected for porosity quantification, (**b**) Example of areas selected for the image analysis.

The distribution of porosity as a function of the distance from the interface was estimated based on the SEM images using the image analysis software from ImageJ. All images were taken at a magnification of 1000 times at various locations and distances from the transition zone, as shown in Figure 2a. Each scanned area had dimensions of 229 × 208 µm, which covered nine images taken at 1000× magnification and was divided into 10 horizontal strips with a width of 20 µm each. A similar approach has also been used by others [34]. Each strip had approximately a length of 228 µm and a width of 20 µm. Three rectangle areas with dimensions 15 × 8 µm were selected on each strip. The selected areas in the interface are identified as 1.1, 1.2 and 1.3 respectively on both side of the interface. The selected areas in the second strip are identified as 2.1, 2.2 and 2.3 as shown in Figure 2b. In the selection process, areas containing aggregates or large air voids were excluded. The used procedure enabled to elimination from the analysis ITZs formed in the bulk cement paste–aggregate zone.

The Gaussian filter was applied to each of the selected areas to reduce the noise and enhance the contrast between the various hydration phases and pores [31]. The threshold level of the grey level histogram corresponding to pores was chosen based on the procedure proposed elsewhere [33]. An example of the used segmentation process is shown in Figure 2b.

Mechanical properties were assessed based on compressive and flexural strength measurements at 28 days of sampling. The 28-day compressive strength was determined by following the SS-EN 12390-3 “Testing hardened concrete–Part 3: Compressive strength of test specimens” [35]. For each measurement, a set of three specimens having dimensions of 100 × 100 × 100 mm^3^ was used. The 28-day flexural strength was determined by following the SS-EN12390-5 “Testing hardened concrete–Part 5: Flexural strength of test specimens” [36]. For each measurement, a set of three beams having dimensions of 100 × 100 × 500 mm^3^ was used.

## 3. Test Results and Discussions

### 3.1. Fresh Concrete Properties

The recorded slump and slump flow values varied between 25 and 720 mm. The UHPC without fibers achieved the highest slump flow of 720 mm, while the addition of short steel fibers lowered that value to 620 mm. The normal strength concrete had a slump of only 25 mm, as shown in Table 4. The difference in the slump values is related to the amount of superplasticizer added to the concrete mix but also to the high proportion of fine grains in the UHPC mix. On the other hand, the presence of steel fiber decreases the flowability of the material because the presence of steel fibers increases the movement resistance of the particles in fresh UHPC through friction and cohesive forces [37,38]. Composite samples containing two types of concretes were casted either in horizontal or vertical layers. Casting in vertical layers aimed to simulate the manufacture of walls, column beams, and other vertical elements. While casting in horizontal layers aimed to simulate the production of slabs and beams.

As described earlier, while casting vertical layers, a temporary plate was placed in the mold to separate the two types of concrete during their casting. Shortly after casting, the plate was removed to enable a wet-on-wet connection. Generally, the visual examination of the produced samples after their demolding did not reveal any signs of intermixing. This trend was observed even when the casted concretes had very different workability, for example, fluid-like UPHC and a rather stiff NSC. In all investigated cases the interface appeared as a nearly straight line. Similar results, when casting wet-on-wet, have been observed by others [39,40].

**Figure 3:**
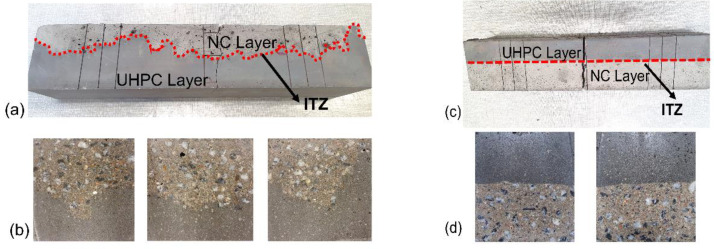
A horizontal layer of hybrid concrete casted in two different arrangements. (**a**) Fluid concrete in the bottom, (**b**) Cross section of the beam when fluid concrete in the bottom, (**c**) Fluid concrete on the top, (**d**) Cross section of the beam when fluid concrete is on top.

The situation became significantly more complex when the two layers were casted horizontally to simulate the production of slabs or beams. Here, the fluid UHPC concrete has been cast first, followed by the placement of a stiffer NC concrete, which is shown in Figure 3a. A practical application of the hybrid concrete by using the fluid concrete in the bottom, e.g., UHPC could be the bottom of a bridge deck. Similar arrangements of the layers were also performed in previous research, where the flowable concrete was placed on the bottom. An improvement in the load-bearing capacity was related to enhanced stiffness and increased cracking resistance of the hybrid concrete [41,42]. Other studies have concluded that a hybrid structure, i.e., UHPC bottom layer and NC upper layer had an improvement in durability. Less aggressive substances migrated into concrete due to denser microstructure and lower permeability of the UHPC [22,43,44]. In this set up the visual examination of the lateral surfaces after demolding showed large variability in the thickness of each layer, as shown in Figure 3b. The stiffer NSC concrete penetrated down through the fluid-like UHPC by up to 30 cm and pushed it up at different locations. The decrease of the ultimate thickness of the external layer made of a durable UHPC, even possibly to a nearly zero value, could lead to serious durability problems, e.g., corrosion of reinforcement. This could occur if only the external layer is designed to resist exposure to environmental factors. While the internal layer has a sufficient load-bearing capacity but at the same time insufficient durability. In another setup, a casting of the fluid-like UHPC on the top of already cast stiffer NSC concrete has been simulated, as shown in Figure 3c. In that case, the UHPC did not tend to penetrate the NC concrete, which resulted in the formation of a nearly straight-line interface, which is shown in Figure 3d. These results indicate a possibly demanding technological problem of placing horizontal layers of two types of concretes having high workability.

### 3.2. Microstructure and Phase Composition

**Figure 4:**
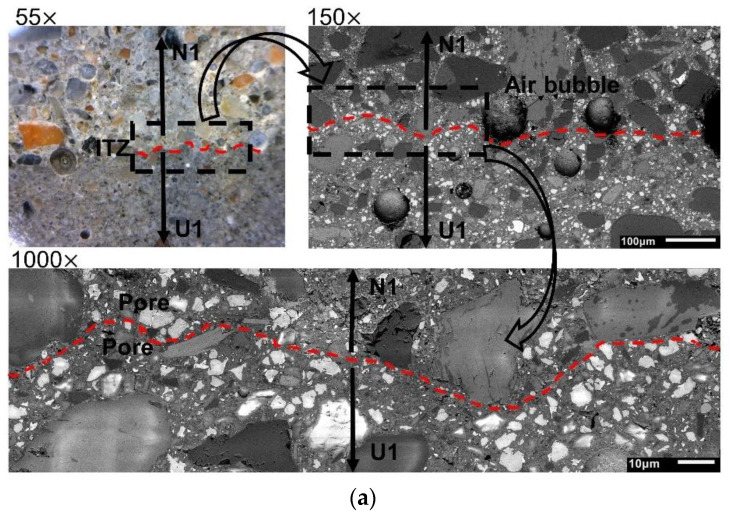

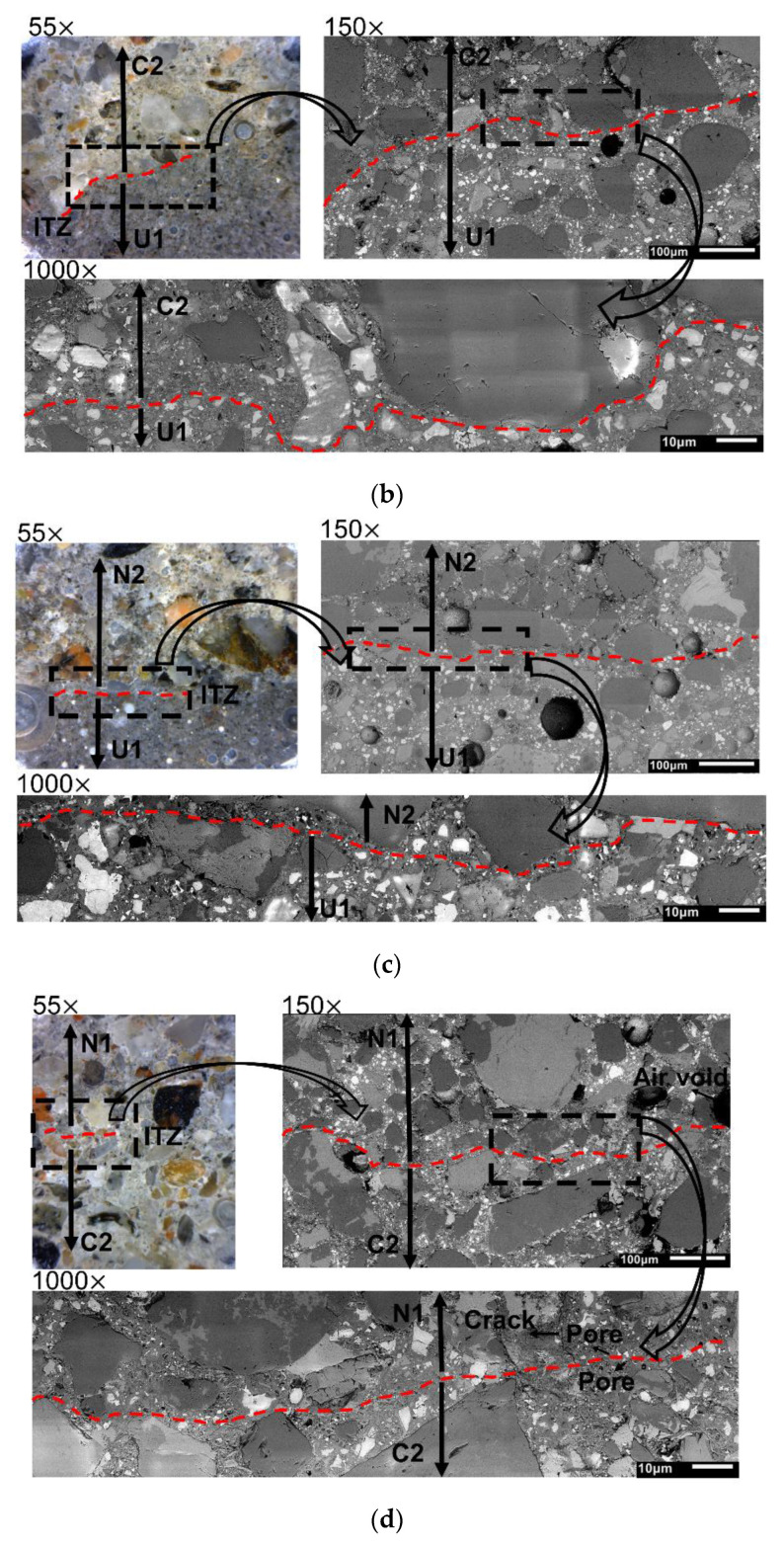
Transition zone observed in hybrid concrete at 55× magnification (digital light microscope) and at 150× and 1000× magnification (SEM): (**a**) U1N1, (**b**) U1C2, (**c**) U1N2, (**d**) N1C2.

**Figure 5:**
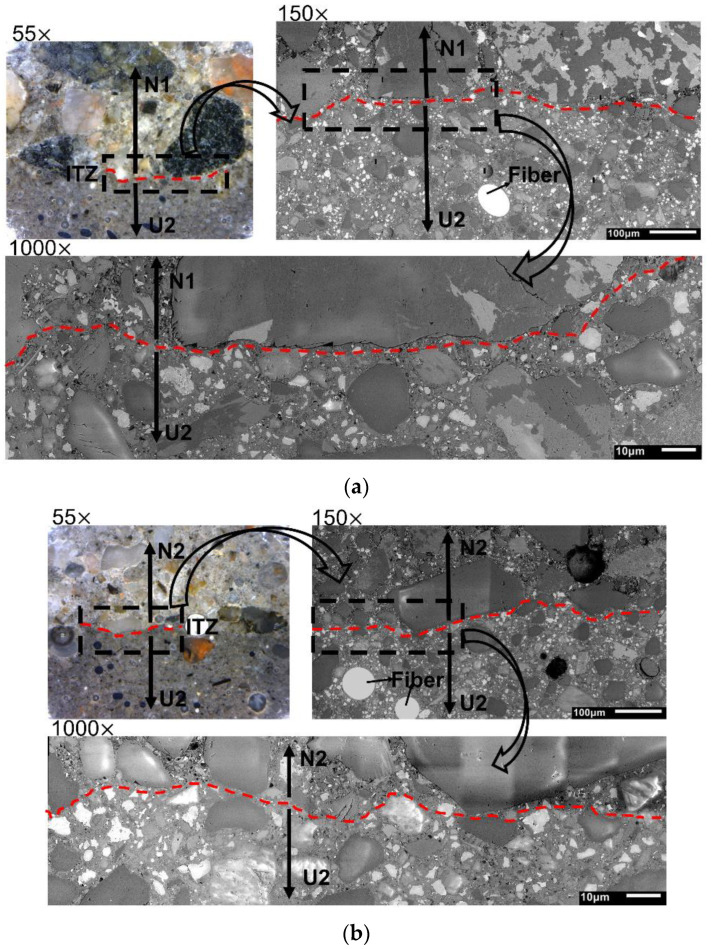
Transition zone observed in hybrid concretes at 55× magnification (digital microscope) and at 150× and 1000× magnification (SEM): (**a**) U2N1, (**b**) U2N2.

Examples of combined images obtained from digital microscope and SEM are shown in Figure 4 and Figure 5. As described earlier, the initial analysis has been performed at low magnification (55×) using images from the digital microscope. These color images enabled for localizing of the transition line based on different colors of the hardened binder matrixes. The UHPC and HPC concrete containing condensed dark grey silica fume appeared darker in comparison with the normal strength concrete (NSC). The slag containing concretes (N1C2) appeared slightly lighter in comparison with the normal concrete. This enabled a selection of areas containing the transition zone to perform their analysis at larger magnifications using the SEM. The 150× magnification has been used to generally overview the morphology, localize aggregates, and main hydration phases, but especially including the distribution of the C-S-H gel.

The results showed a generally higher amount of unhydrated Portland cement and a lack of coarse aggregates in the case of the UHPC mixes. Both are related to the used mix designs, i.e., higher cement content, the addition of limestone, and lower water-to-cement ratio in comparison with the normal concrete.

Two types of transition zones have been identified. The first type was a regular ITZ forming between binder matrix and aggregates, which is known from several earlier studies [45]. In the case of the studied hybrid concrete, there is a possibility that ITZ can form between aggregate and binder matrix originating from the same or different concretes. The second type of transition zone, unique for the developed hybrid concrete, has formed between binder matrixes originating from different concretes.

To study in more detail all types of observed ITZs present in the hybrid concrete, SEM studies at 1000× magnifications have been applied. The investigation focused on microstructure, especially porosity, crack formation, and phase composition.

**Figure 6:**
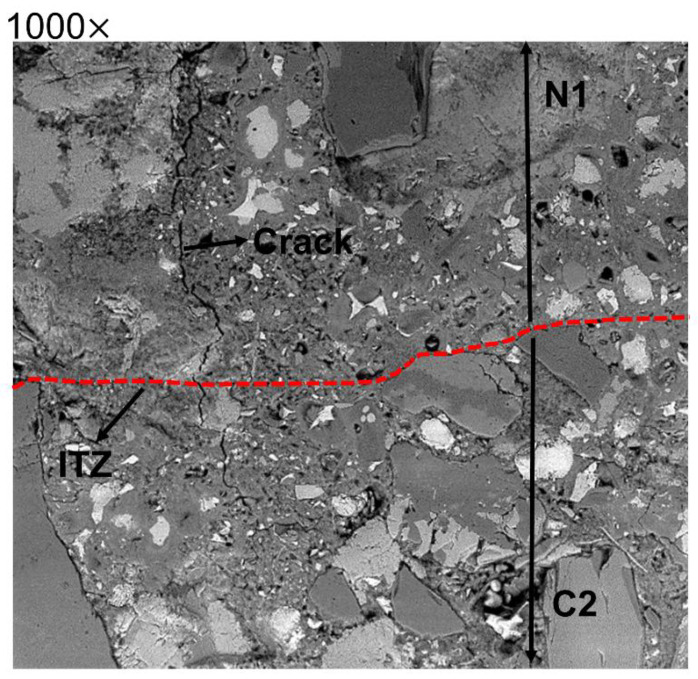
SEM images at 100× of N1C2 where the crack is formed on the N1 matrix and continues to the C2 matrix.

The porosity of the binder matrix was determined directly in the interface zone and in the bulk binder. The bulk binder was assumed not to be affected by the hybrid ITZ and to be located at least 100 µm from the identified interface line. No visible cracks were observed in the interface except for the hybrid N1C2 made of the normal concrete and the blast furnace slag concrete, as shown in Figure 6. The formed microcrack extended from the bulk binder zone of the N1 concrete and continued to the bulk binder zone of the C2 concrete. Microcracking could be related to differences in developed autogenous and drying shrinkages. Earlier studies showed contradicting results indicating either increased or decreased shrinkage of concretes containing GGBFS, as here in the mix C2, which contained 50 wt% of a high MgO content GGBFS [46,47,48].

**Figure 7:**
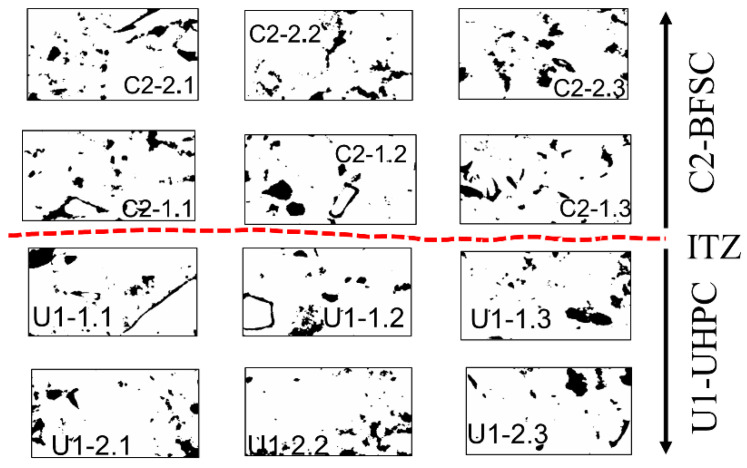
Pore segmentation of the selected area presented in Figure 2.

**Figure 8:**
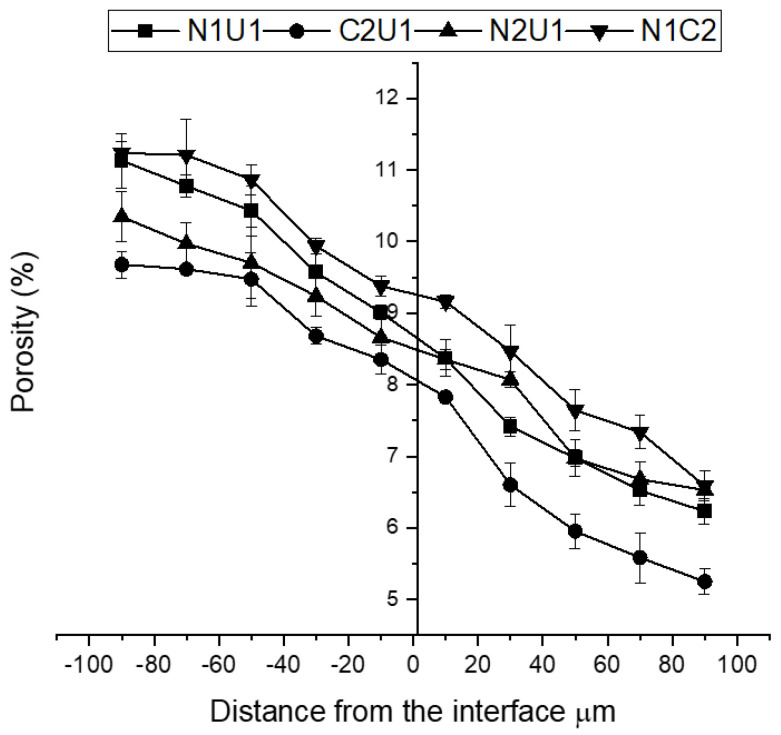
Average porosity presented as a function of the distance from the ITZ.

To obtain the porosity distribution on the interface, the segmentation of images was performed using the ImageJ software, as shown in Figure 7**.** The black areas represent pixels associated with pores. On each studied strip, having a width of 20 µm, three different areas were selected and used to quantify the porosity, which is shown in Figure 2b. The analyzed areas were located at distances between 0 to 100 µm from the interface line and localization was established based on the study performed with the digital microscope. The interface is marked as a red line. An average porosity value was calculated for each analyzed area and the results are shown in Figure 8. Negative X values correspond to the porosity values for the inner layer N1, N2, and C2 while positive X values correspond to the outer layer U1/C2. Generally, the normal strength concrete (N1), having a water-to-cement ratio of 0.5 showed the highest, while the UHPC had the lowest porosity, 11.13% and 5.3%, respectively. The normal strength concrete (N2) with the water to binder ratio of 0.65 had a total porosity of 10.4%, while the blast furnace slag concrete (C2) reached 9.7%. The calculated average porosity determined in each of the analyzed squares tended to increase nearly linearly in the cross-section of the analyzed hybrid ITZ. These trends contradicted somehow earlier observations of the ITZ forming around aggregate particles. In that case, the porosity generally tended to increase towards the aggregate surface and could reach a width of well over 100 µm [31,49,50,51].

The porosity tended to be higher in the inner layer (N1, N2, C2) compared to the outer layer (U1, C2) of the hybrid concrete, as shown in Figure 8. The porosity of UHPC and BFSC in the outer layer tends to decrease. The lower W/C ratio and the presence of the silica fume in the case of the UHPC mix were the main contributing factors. The lower measured porosity of the mix C2 containing 50 wt% of GGBFS can be related to the latent hydraulic reactions, which densified the microstructure due to the formation of more C-S-H. None of the studied hybrids showed a visible increase of the porosity directly in the hybrid ITZ. Furthermore, the porosity transitioned smoothly between the two concretes. The same trend was observed in another study where the porosity decreased from NC to UHPC casted wet–on-wet [52]. Casting wet-on-wet most probably enabled intermixing of both concretes in the transition zone creating a hybrid concrete mix. The width of that zone could be estimated to vary between 60 to 160 μm depending on the flowability and cohesiveness of the used concretes. More cohesive and/or stiffer mixes will presumably intermix less and form a narrower hybrid ITZ. It can be also hypothesized that the properties and mix composition of that hybrid material change gradually throughout the cross-section of the hybrid transition zone from one mix to the other. This smooth transition prevented the formation of microcracks, except for the one mix described earlier. It enabled a smooth increase of stresses developing due to the hydration of Portland cement throughout the cross-section of the transition zone. The developed gradient in most cases was below the maximum tensile stresses thus preventing microcracking.

**Figure 9:**
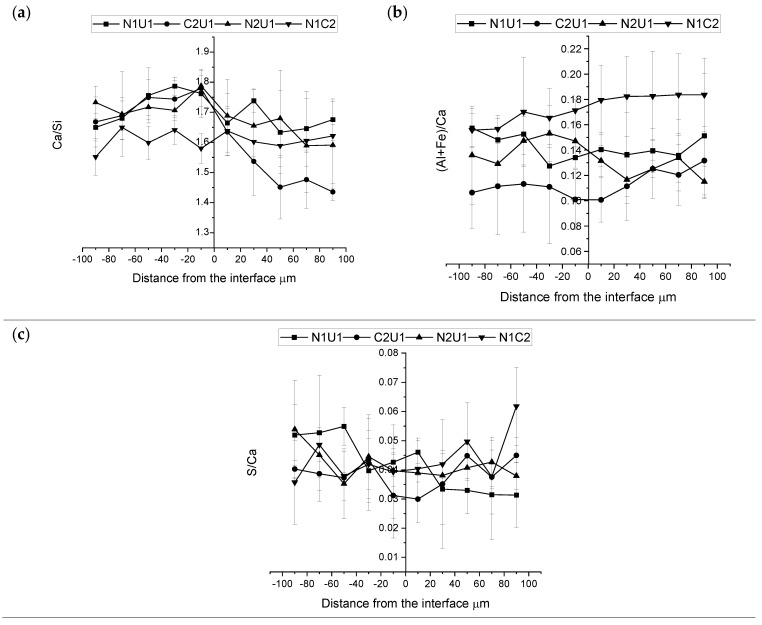
Average atomic ratio and its corresponding standards deviations of hybrid concrete (U1N1, U1C2, U1N2, N1C2) (**a**) Ca/Si, (**b**) (Al+Fe)/Ca, (**c**) S/Ca.

**Figure 10:**
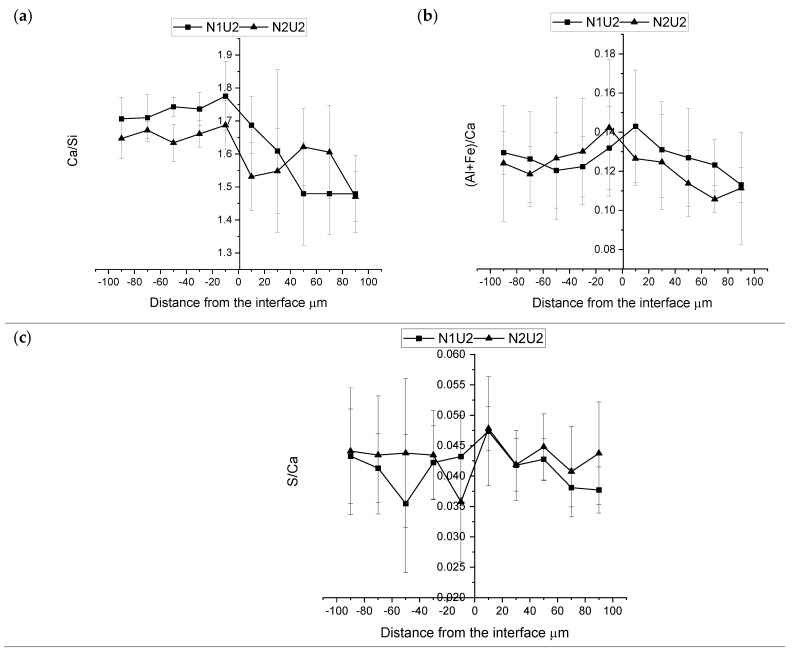
Average atomic ratio and its corresponding standard deviation of hybrid concrete (U2N1, U2N2) (**a**) Ca/Si, (**b**) (Al+Fe)/Ca, (**c**) S/Ca.

The phase composition of the hybrid ITZ was determined by SEM-EDX. All analyzed spots were localized in areas determined as C-S-H based on their grey levels as seen on the SEM-BSE images. Three atomic ratios were calculated and analyzed, i.e., Ca/Si, Al+Fe/Ca, and S/Ca. The results are summarized in Figure 9 and Figure 10 where the value of the inner layer is presented on the negative side of the *x*-axis and the value of the outer layer on the positive side of the *x*-axis.

The calculated Ca/Si atomic ratio has been generally higher for the normal strength concretes (N1 and N2) in comparison with the UHPC mixes (U1 and U2) when measured in the bulk binder matrix. These results are related to a lower amount of the formed Portlandite due to the pozzolanic reactions of SF to produce C-S-H gel [53]. The Ca/Si distribution in the hybrid transition zone varied significantly between the studied combinations of concretes. However, in general, the ratio tended to increase for the UHPC concretes with a lower water-to-cement ratio closer to the interface. On the contrary, the normal strength concrete having a higher water-to-cement ratio showed mostly no change or only a slight increase. The hybrid system composed of UHPC (U1), and normal strength concrete (N2) had higher Ca/Si atomic ratios closer to the interface and lower values away from it. The created peak corresponded well to the results which have been observed in ITZs formed close to coarse aggregates [31,33]. Similar trends and formations of the Ca/Si ratio peak at the interface have formed for the hybrids composed of the UHPC (U1) and the BFS concrete (C2), as well as for the combination of N1 with U1. The observed higher Ca/Si ratio can be directly related to the formation of Portlandite on the interface due to a locally higher water-to-cement ratio, similar to the paste/aggregate ITZ. The (Al+Fe)/Ca and S/Ca atomic ratios were determined to study the formation of AFt and AFm phases [33,54]. The results could indicate that no enhanced formation of AFt or AFm phases occurred in any of the studied hybrids concretes. Blast furnace slag concrete mix (C2) showed the highest ultimate (Al+Fe)/Ca atomic ratio. The ratio was even higher in comparison with the normal concrete (N1). The ratio increased for the N1 concrete towards the interface. The higher ratio can be related to a lower amount of Ca ions caused by the presence of the GGBFS. The GGBFS has two major effects on the hydration of Portland cement, i.e., pozzolanic and latent hydraulic reactions [55,56]. Some studies have indicated that the ultimate effect is more related to the latent hydraulic reaction [57,58]. Spaces originally occupied by slag or Portland cement tended to be filled with C-S-H having altered chemical composition. The combined TEM (transmission electron microscopy) and microprobe analyses indicated intermixing with Mg and Al-rich hydroxide phases [59]. The Ca/Si atomic ratio tended to be unchanged for binder combinations having lower replacement levels and some reduction of that ratio at higher levels. The pH-buffering capacity has also been indicated to be yet another reason for the observed lower Ca/Si ratio of the C-S-H phase [59]. The presence of GGBFS also triggered the formation of the calcium aluminate hydrate (C_3_AH_6_) [60]. Thus summarizing, all listed trends can explain the observed higher measured (Al+Fe)/Ca atomic ratio for the blast furnace slag mix C2. The S/Ca atomic ratio appeared to be similar for all mixes.

### 3.3. Mechanical Properties

Mechanical properties were determined based on compressive and flexural strength measurements. The flexural strength was assessed for two casting arrangements, i.e., vertical, and horizontal, as shown in Figure 1. Beam samples having the vertical arrangement were used to directly assess the bond strength between concretes. Samples casted in the horizontal arrangement aimed to simulate slabs or columns. In these cases, the UHPC concrete would provide enhanced protection against environmental exposure but would also enhance flexural strength. Previous studies showed a significantly higher load-bearing capacity of NSC elements with a cast as wet-on-dry UHPC overlay [19].

**Figure 11:**
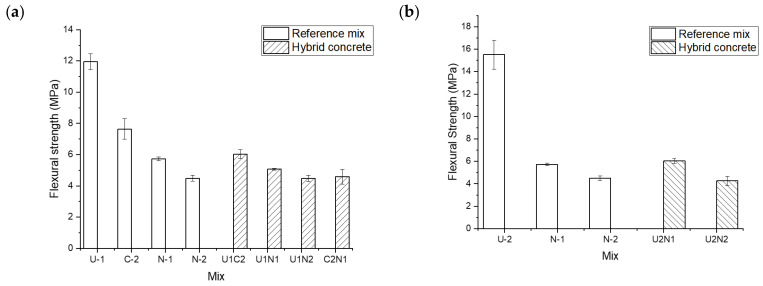
Flexural strengths of hybrid concrete casted in the vertical arrangement; (**a**) combination with UHPC (U1), (**b**) combination with UHPC containing steel fibers (U2).

**Figure 12:**
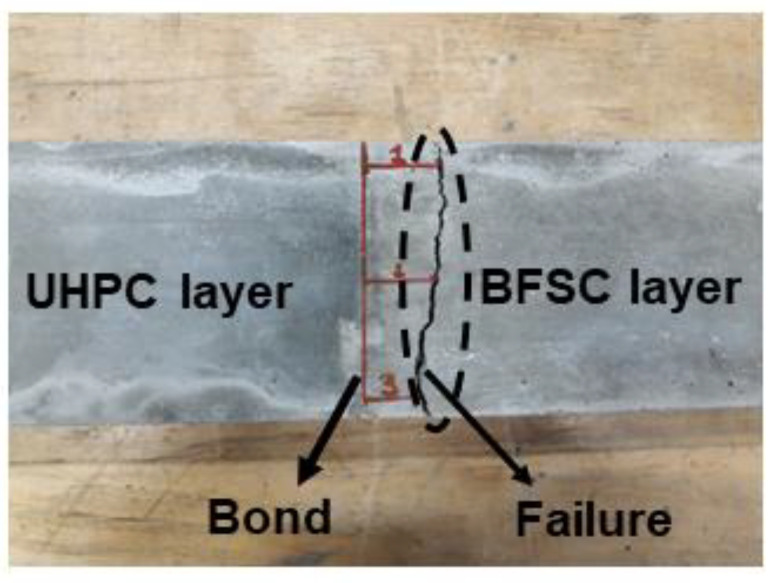
Failure mode of the vertically casted hybrid concrete (U1C2) after three-point bending test.

The results of the three-point bending tests performed on samples casted in the vertical arrangement for the UHPC mixes without (U1) and with steel fibers (U2) are shown in Figure 11. The highest ultimate bending strength of 15.5 MPa was measured for the reference beams made of UHPFRC concrete with steel fibers. The reference UHPC beams without fibers reached 12 MPa. All reference beams made of the normal strength concrete showed significantly lower bending strengths, which reached 7.8, 6, and 4.3 MPa for the C2, N1, and N2 mixes, respectively. The high flexural strength values of the UHPC can be directly related to the low water-to-binder ratio, better packing density, lower porosity, and the presence of silica fume [7,61,62,63]. Samples made of concretes containing BFS (C2) showed a slightly higher flexural strength of 7.8 MPa, which could be directly linked with the latent hydraulic and pozzolanic activities of slag [64,65,66,67]. The failure in bending of beams with the vertical arrangement of layers occurred always in the weaker concrete away from the hybrid ITZ, which is shown in Figure 12. The measured strength values varied between 6 and 4.3 MPa, which were approximately equal to the flexural strengths of N1, N2, and C2 normal strength concretes. These results, in combination with SEM-EDX results, further confirmed the formation of a hybrid ITZ, which is denser and stronger than the weaker concrete. Furthermore, it can be concluded that the hybrid ITZ is generally stronger in comparison with a regular ITZ formed around coarse aggregates. The results also indicated that the hybrid ITZ tended to contain a higher amount of C-S-H over the usually observed increased amount of Portlandite [31]. Similar results were presented by other researchers from studies focusing on casting hybrid concrete by using the wet–on–wet method. The failure occurred along the interface during the splitting tensile strength test, and the crack propagated into a thin layer of the weaker concrete (NC) [25,52].

**Figure 13:**
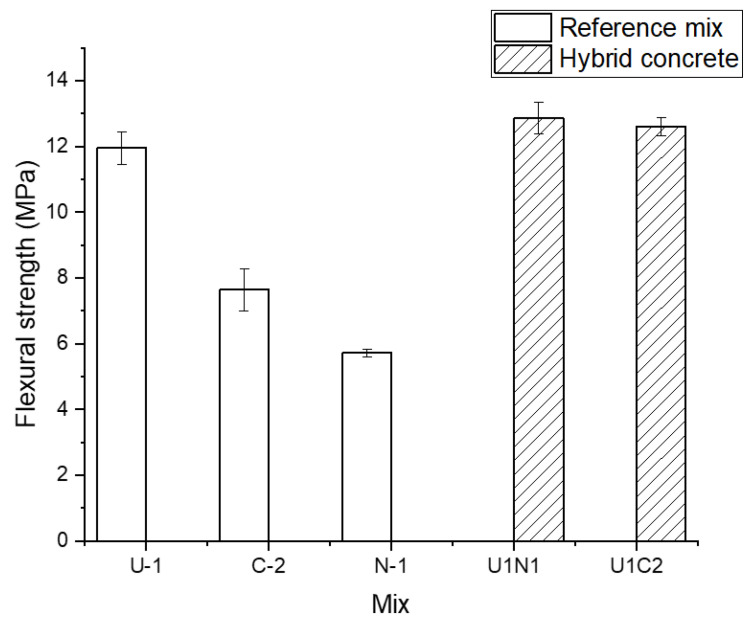
The 28-day flexural strength results of test beams casted in two horizontal layers. In both cases, the UHPC concrete (U1) was located at the bottom of the bent beam.

Bending test results for the hybrid concretes beams cast in horizontal layers are shown in Figure 13. In both studied cases the stronger UHPC (U1) concrete was located on the bottom of the test beam. The top layer has been cast using the normal strength concrete (N1) and concrete without the GGBFS (C2). The results showed in both cases the highest 28-day flexural strength values of around 12 MPa, which equaled the strength of the UHPC (U1) concrete. This occurred despite the lower flexural strength of the normal concrete N1 vs. the slag concrete (C2). None of the tested hybrid samples showed any signs of delamination between the layers, thus further confirming the excellent bond developed in the hybrid ITZ. The main technological problem with such an arrangement of layers is, the describer earlier, the possible penetration of the stiffer concrete into the more fluid concrete during casting. In an extreme case, as shown in Figure 3b, the actual thickness of the bottom layer could be significantly reduced thus leading to a decreased flexural strength. Consequently, the production technology of elements using fluid UHPC as the bottom layer in slabs or beams must be developed to prevent such fluctuations of thickness. The thickness of the bottom layer is of importance for the loading carrying capacity of the hybrid beam. since increasing the loading carrying capacity of the hybrid beam. Yalcinkaya [24] concluded that increasing the thickness of the UHPC layer from 15 mm to 30 mm in the tension zone led to the load–carrying capacity increment ratio of 20% and 34.6% respectively for the hybrid concrete (UHPC-NC) layered horizontally.

**Figure 14:**
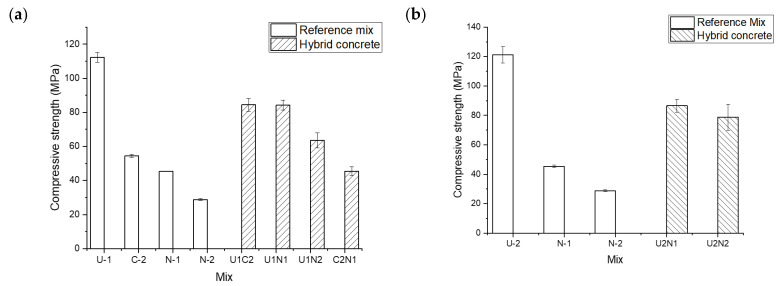
The 28-day compressive strength of hybrid concretes; (**a**) in combination with UHPC (U1) (**b**) in combination with UHPC containing steel fibers (U2).

The 28-day compressive strength of hybrid concretes was determined using vertically cast cubes, Figure 1c. The obtained results are shown in Figure 14. The highest compressive strength of 121.2 was measured for the reference sample made of UHPFRC. The reference sample of UHPC without fibers reached 112.2 MPa. All the reference samples made of the normal strength concretes showed a significantly lower compressive strength, which reached 54.5, 45.5, and 28.8 MPa for the C2, N1, and N2 mixes, respectively. The higher compressive strength value of UHPC can be related to its denser microstructure and lower porosity [7,68]. Furthermore, the addition of steel fibers to the UHPC mix could restrain the initiation and propagation of the vertical cracks in compression which could result in a higher compressive strength [69,70]. The presence of BFS (C2) enhanced the compressive strength due to the pozzolanic reaction [71,72]. Generally, the recorded values were lower than the stronger concrete and higher than the weaker concrete. This could be related to the same thickness of vertical layers, but also originate from the pozzolanic reactions of slag and silica fume. No delamination was observed in any of the test samples.

### 3.4. A Framework of ITZ and Mechanical Properties

The interfacial transition zone formed between two different types of concrete is the key factor, which determines the bond strength and thus the mechanical properties and durability of the casted element structure. The casted interface in the wet–on–wet method when both concrete is poured simultaneously ensured a good bond between the two layers, Figure 3c. The UHPC showed the lowest porosity due to the very low water–binder ratio when compared with the NC and BFSC. In the early stage of the wet–on–wet casting, a small amount of water migrated from the inner layer (NC, BFSC) of the bond to the outer layer (UHPC). The migration of ions between two layers of the hybrid ITZ, based on Fick law [73] occurs from the high concentration of UHPC to the low concentration of the NC/BFSC. More hydration products were formed, which resulted in a denser transition-bonding zone. In this zone, the hydration product and porosity tended to gradually transit from the inner layer to the outer layer. The good bond formed between these two layers was also confirmed by the flexural strength test results. Generally, the failure occurred at a certain distance from the interface of the inner layer (NC/BFSC), which confirmed that the formed hybrid ITZ is denser, and stronger compared to the weaker concrete.

More research is needed to understand how different properties, e.g., workability or density affect the width of ITZ. Moreover, the used casting technique has an important role in the formed ITZ. For example, variation in the duration of the applied vibration between casted layers should be considered.

The hybrid element casted as the wet–on–wet can be a good substitute for the wet–on–hard method. This is due to the good ITZ formed between the layers but also due to the shorter time of the manufacture. There are several practical applications of the hybrid elements such as floors, bridges, columns, and decks that are used to be repaired by the wet–on–hard method to achieve a long life lasting of the structures.

## 4. Conclusions

Hybrid concretes combining two different types of concretes have been studied. All samples have been produced using the wet-on-wet method. The following conclusions have been formulated:

○ Different workability of combined concretes can result in an uneven interface leading to a lower mechanical strength, especially in the case of horizontally cast layers.
○ The ITZ formed between two different concretes cast as wet-on-wet differed substantially from the regular ITZ known to form around coarse aggregate particles where the porosity in this area shows a gradual variation. According to the porosity variation, the width of this area is approximately 35 µm.
○ The binder matrix microstructure and the phase composition tend to transition gradually within the hybrid ITZ.
○ The observed gradual transition presumably prevented the formation of microcracks due to differential absolute shrinkage values.
○ The very dense, homogenous, crack-free hybrid ITZ led to the development of excellent mechanical properties. Since the C-S-H gel is mainly responsible for stiffness and strength, the interface of the hybrid concrete has a higher bonding property. None of the observed failures occurred in the hybrid ITZ during flexural strength.

## Figures and Tables

**Table 1:** Chemical composition of the material used.

	CaO	SiO_2_	Al_2_O_3_	Fe_2_O_3_	MgO	Na_2_O	K_2_O	SO_3_	Cl	LOI
Cement I 42.5 N	63.30	21.20	3.40	4.12	2.20	0.18	0.56	2.70	<0.01	2.50
BFS	30.3	34	11.6	0.291	12.1	0.531	0.811	-	-	−0.9
Silica fume	1	≥85	1	1	1	0.5	1.2	2	0.3	4
Quartz	99.6	-	0.25	0.02	-	-	-	-	-	0.15
Sand (B15, B35)	-	90.5	4.9	0.5	-	1.2	2	-	-	-

**Table 2:** Mix composition.

Ingredient (kg/m^3^)	NSC (N1)	NSC (N2)	BFSC (C2)	UHPC (U1)	UHPFRC (U2)
**Cement (Cem I 42.5N)**	400	360	200	680	664
**BFS**			200		
**Silica Fume 920D**				136	132.8
**Limestone filler**				680	664
**Quartz filler**			92	68	66.4
**Sand–B15**	358	337	92	238	232.4
**Sand–B35**				238	232.4
**Aggregate 0–4**	1254	1179	1106		
**Aggregate 4–8**	179	168	553		
**Steel fibers 7 mm**					66.4
**Steel fibers 13 mm**					99.6
**PCE-superplasticizer**	3	0.9	3	34	33.2
**Air (%)**	2	2	2	4	4
**w/c**	0.45	0.65	0.4	0.3	0.3

**Table 3:** Used combinations of concretes.

Inner Layer →	NSC (N1) (w/c = 0.45)	NSC (N2) (w/c = 0.65)	BFSC (C2) (w/c = 0.4)
**Outer layer ↓**			
**NSC (N1) (w/c = 0.45)**			N1C2
**UHPC (U1) (w/c = 0.3)**	U1N1	U1N2	U1C2
**UHPFRC (U2) (w/c = 0.3)**	U2N1	U2N2	

**Table 4:** Workability of concrete used.

	NSC (N1) (w/c = 0.45)	NSC (N2) (w/c = 0.65)	BFSC (C2) (w/c = 0.4)	UHPC (w/c = 0.3)	UHPFRC (w/c = 0.3)
**Slump** **(mm)**	30 (S)	25 (S)	380 (SF)	720 (SF)	620 (SF)

Note: S-slump height SF-slump flow.

## References

[1] World Business Council for Sustainable Development (2012). The Cement Sustainability Initiative: Executive Brief.

[2] Gartner E. (2004). Industrially interesting approaches to “low-CO2” cements. Cem. Concr. Res..

[3] Wei J., Cen K. (2019). Empirical assessing cement CO 2 emissions based on China’s economic and social development during 2001–2030. Sci. Total Environ..

[4] Kubissa W., Jaskulski R., Reiterman P. (2017). Ecological concrete based on blast-furnace cement with incorporated coarse recycled concrete aggregate and fly ash addition. J. Renew. Mater..

[5] Scrivener K., Martirena F., Bishnoi S., Maity S. (2018). Calcined clay limestone cements (LC3). Cem. Concr. Res..

[6] Jaskulski R., Jóźwiak-Niedźwiedzka D., Yakymechko Y. (2020). Calcined clay as supplementary cementitious material. Materials.

[7] Fehling E. (2014). Ultra-High Performance Concrete UHPC.

[8] Zhang J., Zhao Y., Li H. (2017). Experimental Investigation and Prediction of Compressive Strength of Ultra-High Performance Concrete Containing Supplementary Cementitious Materials. Adv. Mater. Sci. Eng..

[9] Cwirzen A., Penttala V., Vornanen C. (2008). Reactive powder based concretes: Mechanical properties, durability and hybrid use with OPC. Cem. Concr. Res..

[10] Pratama M.M.A., Vertian T., Umniati B.S., Yoh W.H. (2019). Flexural behaviour of the functionally graded concrete beams using two-layers and three-layers configuration. Proceedings of the 2nd International Conference on Green Civil and Environmental Engineering.

[11] Brühwiler E., Denarié E. Rehabilitation of concrete structures using ultra-high performance fibre reinforced concrete. Proceedings of the Second International Symposium on Ultra High Performance Concrete.

[12] Almansour H., Lounis Z. Structural Performance of Precast Prestressed Bridge Girders Built with Ultra High Performance Concrete. Proceedings of the Second International Symposium on UHPC.

[13] Torelli G., Fernández M.G., Lees J.M. (2020). Functionally graded concrete: Design objectives, production techniques and analysis methods for layered and continuously graded elements. Constr. Build. Mater..

[14] Gadri K., Guettala A. (2017). Evaluation of bond strength between sand concrete as new repair material and ordinary concrete substrate (The surface roughness effect). Constr. Build. Mater..

[15] Courard L., Piotrowski T., Garbacz A. (2014). Near-to-surface properties affecting bond strength in concrete repair. Cem. Concr. Compos..

[16] Liu J., Chen Z., Guan D., Lin Z., Guo Z. (2020). Experimental study on interfacial shear behaviour between ultra-high performance concrete and normal strength concrete in precast composite members. Constr. Build. Mater..

[17] Zhang Y., Zhu P., Liao Z., Wang L. (2020). Interfacial bond properties between normal strength concrete substrate and ultra-high performance concrete as a repair material. Constr. Build. Mater..

[18] Varga I.D., Muñoz J.F., Bentz D.P., Spragg R.P., Stutzman P.E., Graybeal B.A. (2018). Grout-concrete interface bond performance: Effect of interface moisture on the tensile bond strength and grout microstructure. Constr. Build. Mater..

[19] Kothari A., Rajczakowska M., Buasiri T., Habermehl-cwirzen K. (2020). Eco-UHPC as Repair Material—Bond Strength, Interfacial Transition Zone and E ff ects of Formwork Type. Materials.

[20] Tayeh B.A., Bakar B.H.A., Johari M.A.M. (2013). Characterization of the interfacial bond between old concrete substrate and ultra high performance fiber concrete repair composite. Mater. Struct..

[21] Tanarslan H.M. (2017). Flexural strengthening of RC beams with prefabricated ultra high performance fibre reinforced concrete laminates. Eng. Struct..

[22] Roesler J., Paulino G., Gaedicke C., Bordelon A., Park K. (2007). Fracture behavior of functionally graded concrete materials for rigid pavements. Transp. Res. Rec..

[23] Rydval M., Čítek D., Kolísko J., Nenadálová Š., Bittner T. (2017). Functionally layered thin slabs made from UHPC and ECC composites. Solid State Phenom..

[24] Yalçınkaya Ç. (2021). A preliminary study on the development of the normal concrete-UHPC composite beam via wet casting. J. Struct. Eng. Appl. Mech..

[25] Hussein L., Amleh L. (2015). Structural behavior of ultra-high performance fiber reinforced concrete-normal strength concrete or high strength concrete composite members. Constr. Build. Mater..

[26] Hussein L., Amleh L., Siad H., Lachemi M. (2018). Effect of very severe sulfate environment on bonded composite concrete system. Constr. Build. Mater..

[27] Hussein L., Amleh L., Amleh L., Siad H., Lachemi M. (2020). Effect of severe chloride environment on the flexural behaviour of hybrid concrete systems. Mag. Concr. Res..

[28] Chu S.H. (2019). Effect of paste volume on fresh and hardened properties of concrete. Constr. Build. Mater..

[29] Abbas S., Nehdi M.L., Saleem M.A. (2016). Ultra-High Performance Concrete: Mechanical Performance, Durability, Sustainability and Implementation Challenges. Int. J. Concr. Struct. Mater..

[30] ICS (2021). Testing Fresh Concrete—Part 8: Self-Compacting Concrete–Slump-Flow Test.

[31] Cwirzen A., Penttala V. (2005). Aggregate-cement paste transition zone properties affecting the salt-frost damage of high-performance concretes. Cem. Concr. Res..

[32] Rossen J.E., Lothenbach B., Scrivener K.L. (2015). Composition of C-S-H in pastes with increasing levels of silica fume addition. Cem. Concr. Res..

[33] Scrivener K.L. (2004). Backscattered electron imaging of cementitious microstructures: Understanding and quantification. Cem. Concr. Compos..

[34] Diamond S., Huang J. (2001). The ITZ in concrete—A different view based on image analysis and SEM observations. Cem. Concr. Compos..

[35] ICS (2020). Testing Hardened Concrete– Part 3: Compressive Strength of Test Specimens.

[36] ICS (2020). Testing Hardened Concrete Flexural Strength of Test Specimens.

[37] Bonen D., Shah S.P. (2005). Fresh and hardened properties of self-consolidating concrete. Prog. Struct. Eng. Mater..

[38] Martinie L., Rossi P., Roussel N. (2010). Rheology of fiber reinforced cementitious materials: Classification and prediction. Cem. Concr. Res..

[39] Brault A., Lees J.M. (2020). Wet casting of multiple mix horizontally layered concrete elements. Constr. Build. Mater..

[40] Torelli G., Lees J.M. (2019). Fresh state stability of vertical layers of concrete. Cem. Concr. Res..

[41] Zhang Y., Zhu Y., Yeseta M., Meng D., Shao X., Dang Q., Chen G. (2019). Flexural behaviors and capacity prediction on damaged reinforcement concrete (RC) bridge deck strengthened by ultra-high performance concrete (UHPC) layer. Constr. Build. Mater..

[42] Zhu Y., Zhang Y., Hussein H.H., Chen G. (2020). Flexural strengthening of reinforced concrete beams or slabs using ultra-high performance concrete (UHPC): A state of the art review. Eng. Struct..

[43] Maalej M., Ahmed S.F.U., Paramasivam P. (2003). Corrosion durability and structural response of functionally-graded concrete beams. J. Adv. Concr. Technol..

[44] Maalej M., Li V.C. (1995). Introduction of Strain-Hardening Engineered Cementitious Composites in Design of Reinforced Concrete Flexural Members for Improved Durability. ACI Struct. J..

[45] Cwirzen A. (2004). Effects of the Transition Zone and Ageing on the Frost Damage of High Strength Concretes.

[46] Hu X., Shi Z., Shi C., Wu Z., Tong B., Ou Z., de Schutter G. (2017). Drying shrinkage and cracking resistance of concrete made with ternary cementitious components. Constr. Build. Mater..

[47] Mizuno H., Iyoda T. Shrinkage characteristics of ground granulated blast furnace slag high content cement. Proceedings of the Life-Cycle Analysis and Assessment in Civil Engineering: Towards an Integrated Vision. In Proceedings of the 6th International Symposium on Life-Cycle Civil Engineering.

[48] Yuan J., Lindquist W., Darwin D., Browning J.A. (2015). Effect of slag cement on drying shrinkage of concrete. ACI Mater. J..

[49] Berodier E., Scrivener K. (2015). Evolution of pore structure in blended systems. Cem. Concr. Res..

[50] Herrmann M., Sobek W. (2017). Functionally graded concrete: Numerical design methods and experimental tests of mass-optimized structural components. Struct. Concr..

[51] Scheydt J., Müller H. Microstructure of ultra high performance concrete (UHPC) and its impact on durability. Proceedings of the 3rd International Symposium on on Ultra High Performance Concrete.

[52] Liu S., He Z., Hu L. (2022). Interfacial microstructure between ultrahigh-performance concrete–normal concrete in fresh-on-fresh casting. Constr. Build. Mater..

[53] Bentur A., Cohen M.D. (1987). Effect of Condensed Silica Fume on the Microstructure of the Interfacial Zone in Portland Cement Mortars. J. Am. Ceram. Soc..

[54] Vivekanandam K., Patnaikuni I. (1997). Transition zone in high performance concrete during hydration. Cem. Concr. Res..

[55] Bougara A., Lynsdale C., Milestone N.B. (2018). The influence of slag properties, mix parameters and curing temperature on hydration and strength development of slag/cement blends. Constr. Build. Mater..

[56] Escalante-García J.I., Sharp J.H. (1998). Effect of temperature on the hydration of the main clinker phasesin Portland cements: Part II, blended cements. Cem. Concr. Res..

[57] Kocaba V. (2009). Development and Evaluation of Methods to Follow Microstructural Development of Cementitious Systems Including Slags.

[58] Luke K., Glasser F.P. (1988). Internal chemical evolution of the constitution of blended cements. Cem. Concr. Res..

[59] Richardson I.G., Groves G.W. (1992). Microstructure and microanalysis of hardened cement pastes involving ground granulated blast-furnace slag. J. Mater. Sci..

[60] Wang K.S., Lin K.L., Tzeng B.Y. (2003). Latent Hydraulic Reactivity of Blended Cement Incorporating Slag Made from Municipal Solid Waste Incinerator Fly Ash. J. Air Waste Manag. Assoc..

[61] Ghasemzadeh Mosavinejad S.H., Langaroudi M.A.M., Barandoust J., Ghanizadeh A. (2020). Electrical and microstructural analysis of UHPC containing short PVA fibers. Constr. Build. Mater..

[62] Cwirzen A. Effects of increased aggregate size on the mechanical and rheological properties of RPC. Proceedings of the Second International Symposium on Advances in Concrete through Science and Engineering.

[63] Cwirzen A. (2007). The effect of the heat-treatment regime on the properties of reactive powder concrete. Adv. Cem. Res..

[64] Yun C.M., Rahman M.R., Phing C.Y.W., Chie A.W.M., Bakri M.K. (2020). Bin The curing times effect on the strength of ground granulated blast furnace slag (GGBFS) mortar. Constr. Build. Mater..

[65] Desta E., Jun Z. A Review on Ground Granulated Blast Slag GGBS in Concrete. Proceedings of the Eighth International Conference on Advances in Civil and Structural Engineering, CSE.

[66] Samad S., Shah A. (2017). Role of binary cement including Supplementary Cementitious Material (SCM), in production of environmentally sustainable concrete: A critical review. Int. J. Sustain. Built Environ..

[67] Suresh K.N.D. Ground Granulated Blast Slag (GGBS) In Concrete—A Review. Proceedings of the Eighth International Conference On Advances in Civil and Structural Engineering.

[68] Ranjan P., Iyer N.R. (2013). Influence of curing regimes on compressive strength of ultra high performance concrete. Sadhana.

[69] Meng W., Khayat K.H. (2018). Effect of Hybrid Fibers on Fresh Properties, Mechanical Properties, and Autogenous Shrinkage of Cost-Effective UHPC. J. Mater. Civ. Eng..

[70] Yang J., Chen B., Su J., Xu G., Zhang D., Zhou J. (2022). Effects of fibers on the mechanical properties of UHPC: A review. J. Traffic Transp. Eng..

[71] Yazici H., Yardimci M.Y., Yiǧiter H., Aydin S., Türkel S. (2010). Mechanical properties of reactive powder concrete containing high volumes of ground granulated blast furnace slag. Cem. Concr. Compos..

[72] Teng S., Lim T.Y.D., Sabet Divsholi B. (2013). Durability and mechanical properties of high strength concrete incorporating ultra fine Ground Granulated Blast-furnace Slag. Constr. Build. Mater..

[73] Chatterji S. (1994). Transportation of ions through cement based materials. Part 1 fundamental equations and basic measurement techniques. Cem. Concr. Res..

